# Screening of miRNA profiles and construction of regulation networks in early and late lactation of dairy goat mammary glands

**DOI:** 10.1038/s41598-017-12297-4

**Published:** 2017-09-20

**Authors:** Zhibin Ji, Zhaohua Liu, Tianle Chao, Lei Hou, Rui Fan, Rongyan He, Guizhi Wang, Jianmin Wang

**Affiliations:** Shandong Provincial Key Laboratory of Animal Biotechnology and Disease Control and Prevention, Shandong Agricultural University, 61 Daizong Street, Taian Ctity, Shandong Province 271018 P.R. China

## Abstract

In recent years, studies related to the expression profiles of miRNAs in the dairy goat mammary gland were performed, but regulatory mechanisms in the physiological environment and the dynamic homeostasis of mammary gland development and lactation are not clear. In the present study, sequencing data analysis of early and late lactation uncovered a total of 1,487 unique miRNAs, including 45 novel miRNA candidates and 1,442 known and conserved miRNAs, of which 758 miRNAs were co-expressed and 378 differentially expressed with *P* < 0.05. Moreover, 76 non-redundant target genes were annotated in 347 GO consortiums, with 3,143 candidate target genes grouped into 33 pathways. Additionally, 18 predicted target genes of 214 miRNAs were directly annotated in mammary gland development and used to construct regulatory networks based on GO annotation and the KEGG pathway. The expression levels of seven known miRNAs and three novel miRNAs were examined using quantitative real-time PCR. The results showed that miRNAs might play important roles in early and late lactation during dairy goat mammary gland development, which will be helpful to obtain a better understanding of the genetic control of mammary gland lactation and development.

## Introduction

Dairy goats are a vital domesticated species in worldwide agriculture and animal husbandry; they are dual-purposed for the production of milk and meat and are primarily utilized for milk production to satisfy the new and diversified consumer demand. Mammary glands, as the sole organ of milk synthesis, secretion and storage, determine the yield and quality of milk. While at different stages of mammary gland development, milk yield and components are regularly varied, reflecting physiological and environmental changes^1^, the majority of mammary gland development occurs postnatally, undergoing cyclical periods of growth, differentiation, lactation, and regression, coordinated with the homeostasis, growth, maintenance and replacement of mammary epithelium cells. Studies have demonstrated that during early lactation in dairy goats, mammary growth may account for approximately 20% of the total number of mammary cells, which primarily reflects the proliferation and differentiation of mammary secretory cells, and the increases in milk production result from an increase in mammary cell number and secretory activity per cell^2–4^. When milk production declines, particularly during the late lactation of the mammary gland, this process is characterized by extensive tissue cell apoptosis or remodelling, including changes in cell population, alveolar structure, and extracellular matrix synthesis^5^. Other studies have also demonstrated that the apoptotic death of mammary epithelial cells and their removal by phagocytes, including both macrophages and epithelial cells, are crucial events during mammary gland involution^6,7^. The changes in milk yield at different stages of mammary gland development primarily reflect the counterbalance of apoptosis and proliferation, as well as the changes in the secretory activity of mammary epithelial cells.

The decline in milk yield following peak lactation in dairy animals has long been a biological conundrum and a cause of considerable lost income for the dairy farmer; thus, considerable effort has been expended in recent years to examine the mechanisms for controlling these biological processes using candidate gene methods^8–10^. Indeed, physiological processes are governed by many genes or regulatory factors acting in concert rather than by only one or a few individual genes^11^. During the last decade, with the rapid development of high-throughput sequencing technology, studies at genome-wide levels, such as transcriptomics, proteomics, and microRNomics^12,13^, have become realized, and high-throughput sequencing technology is now widely used in physiological studies of mammogenesis and lactogenesis^14–17^.

MicroRNAs (miRNAs) are endogenous, non-coding RNAs, first identified in eukaryotes with a length of 18–25 bp in animals, and these molecules are currently acknowledged as important regulators of most biological functions^18,19^. Recent studies have shown that miRNAs, by targeting the 3′ untranslated regions (UTR) of mRNAs, are involved in a wide variety of biological processes, including body development, haematopoiesis, organogenesis, fat metabolism, carcinogenesis, cell differentiation, proliferation, apoptosis and many other processes^20,21^, and the regulatory functions of miRNAs on mammary gland development, physiology and homeostasis, mammary cell proliferation, differentiation, and apoptosis have been observed; Kayo and colleges^22^ showed that miR-212/132 are indispensable for mouse mammary gland development. Hou *et al*.^23^ reported 5, 10-methylenetetrahydrofolate reductase (*MTHFR*) as a central regulator of folate metabolism, and mutations in its 3′UTR, associated with milk yield and milk protein levels, could change the binding activity of has-miR-1266 and has-miR-616; these results indicated that miR-1266 and miR-616 are involved in regulatory functions of milk yield and milk protein synthesis in caprine. Through the generation of a knockout mouse model, Liobet-Navas and colleagues^24^ demonstrated that the miR-424(322)/503 cluster orchestrates the remodelling of the epithelium in the involuting mammary gland, and these authors also showed the regression of secretory acini when the mammary gland is compromised in the absence of miR-424(322)/503, as these molecules orchestrate cell life and death decisions by targeting B-cell lymphoma-2 (*BCL-2*) and insulin-like growth factor 1 receptor (*IGF1R*). In previous studies^25,26^, we compared the differential expression of miRNAs between peak and late lactation and observed that miRNAs are widely involved in the physiological activities of mammary gland development and lactation. This evidence suggests that miRNAs are also involved in mammary cell fate and play an important role in mammary gland development, lactation, involution and the synthesis of milk ingredients^27–29^.

MiRNA sequencing techniques based on the Illumina/Solexa high-throughput sequencing platform have overcome the limitations of miRNA research, facilitating the direct sequencing of specific-sized miRNAs from samples to determine miRNA expression profiles and discover or identify novel miRNAs in organisms without any sequence information. In the present study, two small RNA libraries of early and late lactation were structured and sequenced based on the Illumina/Solexa high-throughput sequencing platform. These results will provide a reference for elucidating the determinants of mammary apoptosis and the factors controlling the dynamic balance between cell proliferation and cell apoptosis/death in these two stages of mammary gland development. Further, these findings could provide a theoretical basis for delaying mammary gland involution, increasing lactation production, and cultivating high-yielding dairy goat breeds.

## Results

### Analysis of small RNAs sequencing data from two libraries

**Figure 1:**
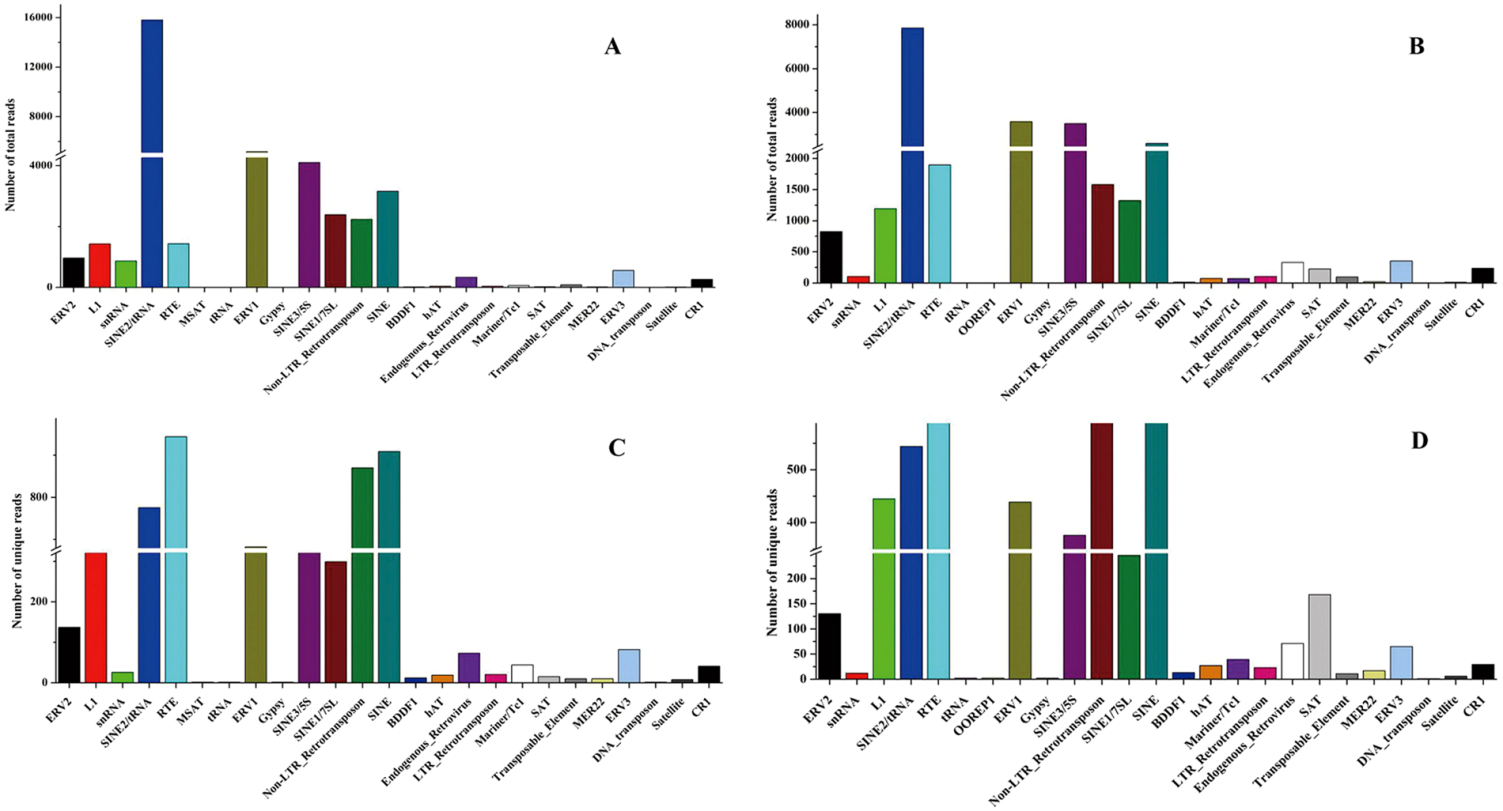
The category of total and unique repeat small RNA (sRNA) sequences in two small RNA libraries aligning in RepBase. (**A**) Different categories of total repeat sequences in early lactation library. (**B**) Different categories of total repeat sequences in late lactation library. (**C**) Different categories of unique repeat sequences in early lactation library. (**D**) Different categories of unique repeat sequences in late lactation library.

**Figure 2:**
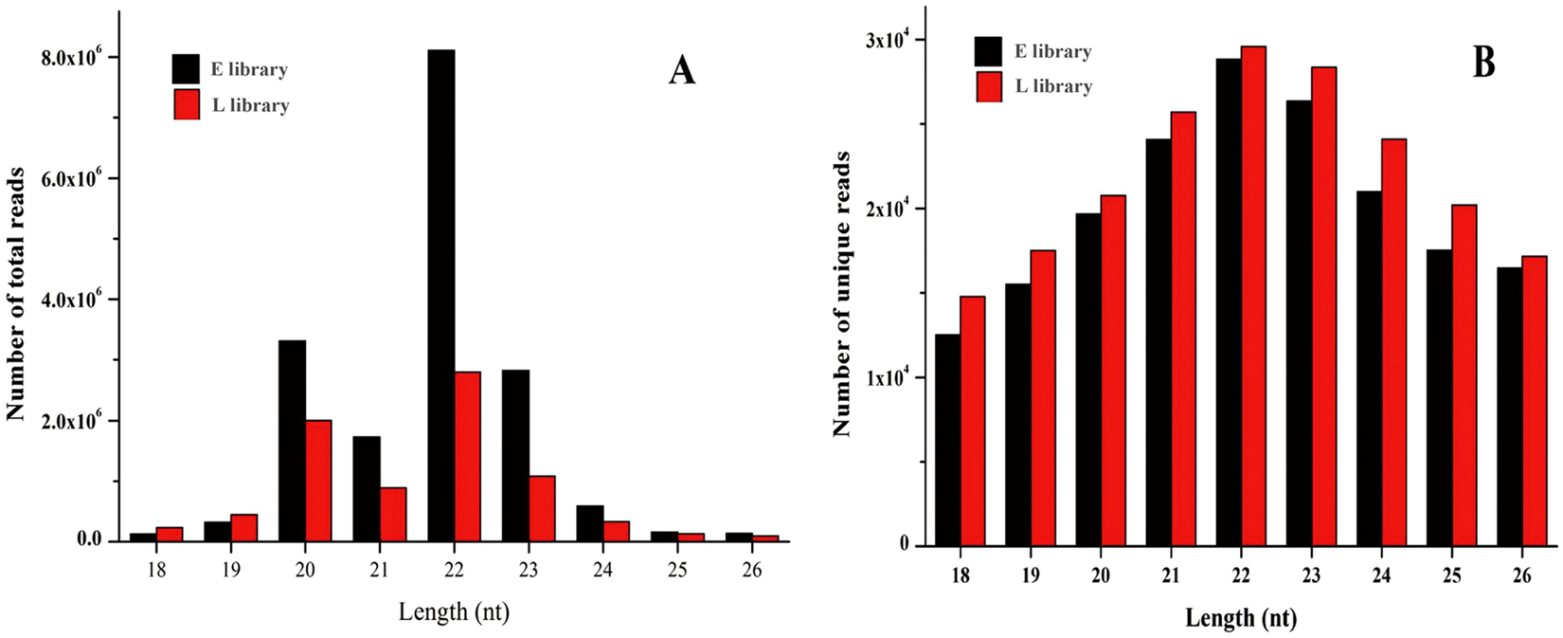
Length distribution of small RNAs in two libraries from mammary gland at early lactation (**E**) and late lactation (**L**) of dairy goat. (**A**) Size distribution of total reads in different lengths (nt) in two libraries. (**B**) Size distribution of unique reads in different lengths (nt) in two libraries.

To identify small RNA (sRNA) from the mammary gland of *Capra hircus*, two small RNA libraries of early lactation (E library, RIN = 8.1) and late lactation (L library, RIN = 8.2) were constructed and sequenced. A total of 18,908,954 and 10,083,672 raw reads, representing 809,387 and 958,982 unique sequences, were generated in E and L libraries, respectively. After removing junk and repeat reads, filtering reads with null 3′ adapters (3ADT) and lengths, and aligning with mRNA and Rfam databases, 85,700 (0.45%) and 62,242 (0.62%) reads were mapped on mRNA, and 314,821 (1.66%) and 164,175 (1.63%) reads were fed back to the Rafm database, including tRNAs, rRNAs snRNA, snoRNAs and other Rfam RNA (Table 1). After aligning with prototypic sequences (repetitive DNA from different eukaryotic species), 38,984 (0.21%) and 25,990 (0.26%) reads were named as repeats, and the categories of these repeat-associated small RNA sequences are shown in Fig. 1. Finally, a total of 17,306,229 and 7,997,385 valid reads accounting for 91.52% and 79.31% of total reads, corresponding to 181,983 and 198,134 unique reads, respectively, ranging from 18 to 26 nucleotides, were obtained from the two libraries and used for further analysis. In the two libraries, the overall length distributions were similar, and the most abundant molecule was 22 nucleotides in length, accounting for 46.85% and 34.98%, followed by 20, 23 or 21 nucleotides; for unique small RNAs, 22 nt was the most abundant category, accounting for 15.85% and 14.93%, respectively, in the two libraries, and 21–24 nt were the main small RNA classes (Fig. 2).

**Table 1:** Statistics of small RNA (sRNA) sequences from E (early lactation) and L (late lactation) libraries.

Type	E library				L library			
	Number of total sRNA	Percentage of total (%)	Number of unique sRNA	Percentage of unique (%)	Number of total sRNA	Percentage of total (%)	Number of unique sRNA	Percentage of unique (%)
Raw reads	18,908,954	100	809,387	100	10,083,672	100	958,982	100
Junk reads	4,227	0.02	2,218	0.27	4,886	0.05	2,107	0.22
Repeat reads	38,984	0.21	5,927	0.73	25,990	0.26	5,693	0.59
3ADT &length filter^*^	1,197,580	6.33	566,670	70.01	1,851,160	18.36	713,666	74.42
Rfam	314,821	1.66	25.791	3.19	164,175	1.63	21,564	2.25
rRNA	163,022	0.86	10,825	0.06	91,082	0.90	9,750	0.10
tRNA	73,643	0.39	6,258	0.03	38,858	0.39	5,391	0.05
snoRNA	43,634	0.23	3,739	0.02	15,082	0.15	2,239	0.02
snRNA	7,751	0.04	1,355	0.01	2,455	0.02	1,063	0.01
other RNAs	26,771	0.14	3,614	0.02	16,698	0.17	3,121	0.03
mRNA	85,700	0.45	31,921	3.94	62,242	0.62	22,094	2.30
Valid reads	17,306,229	91.52	181,983	22.48	7,997,385	79.31	198,134	20.66

^*^Reads lacking three ADTs or with lengths <18 nt or >26 nt were removed. 3ADT: reads with null 3′ adapter. snRNA: small nuclear RNA. snoRNA: small nucleolar RNA. tRNA, rRNA snRNA, snoRNA and other RNAs were obtained by blasting sRNAs against Rfam database.

### Identification of known and novel miRNAs

To identify the miRNAs in dairy goat mammary gland tissues, all mammalian miRNAs in miRBase v.21 were named as background database to identify known, conserved and novel miRNAs. After discarding the repeats by comparing the miRNAs among different species, a total of 1,160 pre-miRNA and 1,491 unique miRNAs with more than 3 reads were identified in the two small RNA libraries (Table 2); among them, 1,083 and 1,117 miRNAs were detected in the E library and L library, respectively, and 758 miRNAs were co-expressed, with 325 miRNAs specific for the E library and 359 miRNAs specific for the L library. There were 397 unique miRNAs, referred to as known miRNAs (Group 1a), in *Capra hircus*, of which 161 sequences were the same as the mature miRNA sequences in miRBase, 178 sequences were different from the existing miRNAs because of the permitted base replacement or missing bases, and 58 sequences were detected at the other arm of pre-miRNAs. A total of 1,045 unique miRNAs and 912 pre-miRNAs were identified as conserved miRNAs among mammals (Group 1b, 2 and 3), and among these known and conserved miRNAs, 3 miRNAs with more than 100,000 reads (has-miR-518a-2-p5, has-miR-6724-2-p3 and ssa-let-7d-1-p3), and 29 miRNAs with more than 10,000 reads were detected in both libraries. For more detailed information, please refer to Supplement Files: Table S2.

**Table 2:** Number of known miRNAs and novel miRNA candidates in E (early lactation) and L (late lactation) libraries.

Groups	Total		E library		L library	
	Pre-miRNA	Unique miRNA	Pre-miRNA	Unique miRNA	Pre-miRNA	Unique miRNA
Group 1a	293	397	219	330	237	358
Group 1b	138	159	118	132	123	137
Group 2	735	829	513	573	528	584
Group 3	54	57	44	47	35	38
Group 4	47	45	44	42	39	41

^*^Group 1a: the reads mapped to miRNAs/pre-miRNAs of *Capra hircus* in miRBase and the pre-miRNAs further mapped to the genome and EST of *Capra hircus*. Group 1b: the reads mapped to mammalian miRNAs/pre-miRNAs (except for *Capra hircus*) in miRBase and the pre-miRNAs further mapped to the genome and EST of *Capra hircus*. Group 2: the reads mapped to mammalian miRNAs/pre-miRNAs (except for *Capra hircus*) in miRBase, the mapped pre-miRNAs not further mapped to the genome, and the reads (and of course the miRNAs of the pre-miRNAs) mapped to the genome. Group 3: the reads mapped to mammalian miRNAs/pre-miRNAs (except for *Capra hircus*) in miRBase and the reads and the mapped pre-miRNAs not mapped to the genome. Group 4: the reads not mapped to mammalian pre-miRNAs in the miRBase but mapped to the genome and EST of *Capra hircus* and the extended genome sequences that may form hairpin structures.

The reads that did not match any of the known mammalian miRNAs/pre-miRNAs were further analysed to identify potential miRNAs according the criteria in Materials and Methods; a total of 45 miRNA candidates (named as novel miRNAs) were identified (Table 2), 34 of which were identified in both E and L libraries, and these miRNAs had more than three reads with lengths ranging from 18 to 25 nt and with free energy ranging from −141.8 to −17.7 kcal/mol. Moreover, 28 novel miRNAs were 5p-derived sequences, and 17 novel miRNAs were 3p-derived sequences. For more detailed information, please refer to Supplement Files: Table S3.

**Figure 3:**
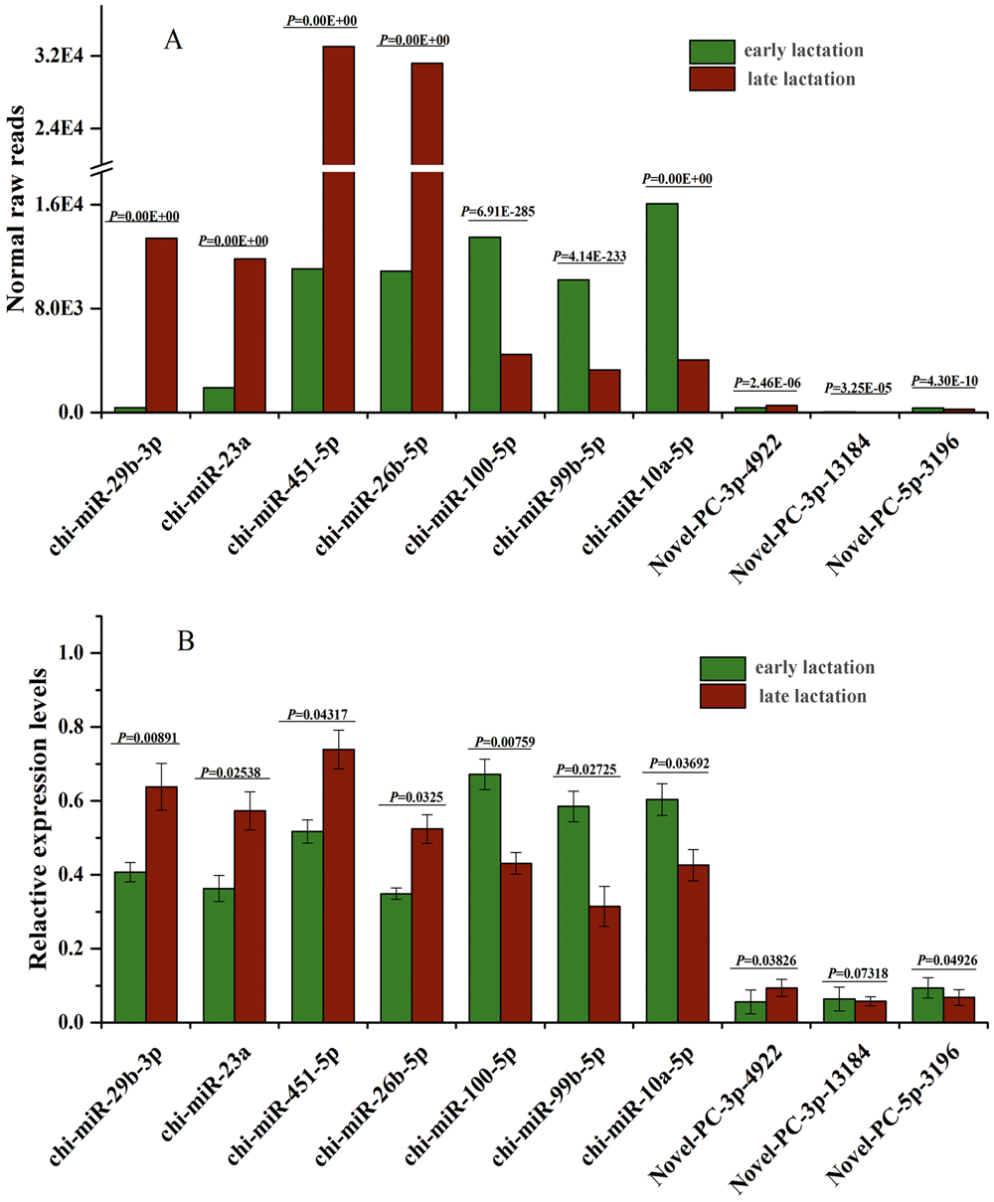
The qRT-PCR validation of the identified miRNAs using Solexa sequencing technology. (**A**) The abundance of the seven known miRNAs and three novel miRNAs in early and late lactation examined using Solexa sequencing. (**B**) The relative expressed levels of the seven known miRNAs and three novel miRNAs tested using qRT-PCR. The relative quantification of expression was calculated using the 2^−ΔΔCT^ method after the threshold cycle (Ct) and normalized to the Ct of U6. The relative expression levels were presented as the 2^−ΔΔCT^ mean ± SE (standard error), and the error bars indicate the standard error of the 2^−ΔΔCT^ mean values. *P* < 0.05 represents significance difference, *P* < 0.01 represents very significant difference.

To detect the authenticity of these identified miRNAs, the expression levels of seven known miRNAs and three novel miRNAs with high abundance in *Capra hircus* were examined in early and late lactation using qRT-PCR (Fig. 3). These results showed that although there were differences in the fold-change values and significance levels, the expression pattern examined using qRT-PCR was consistent with the results of Illumina/Solexa sequencing.

### miRNAs conservation and family analysis

**Figure 4:**
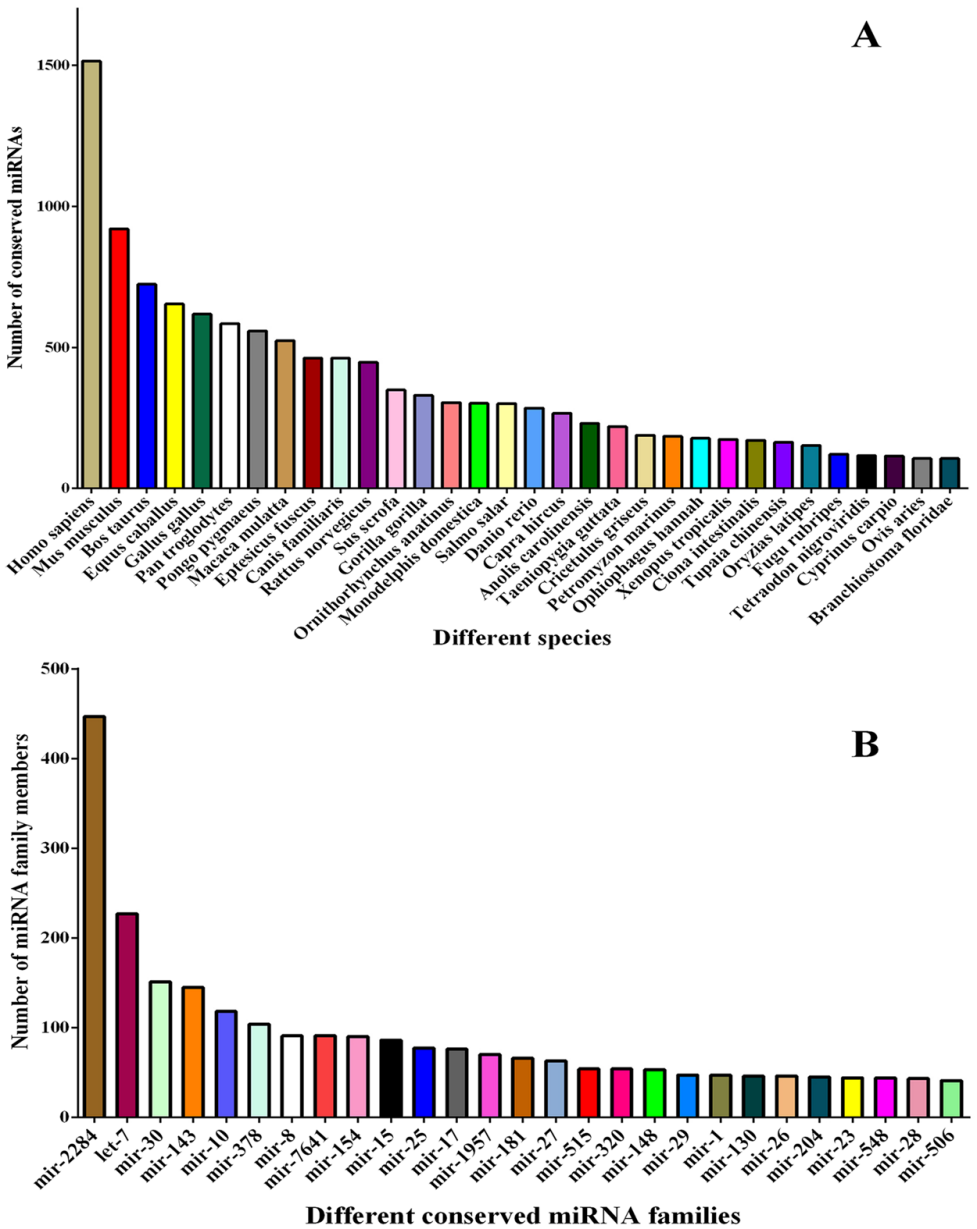
Conservation of the identified miRNAs BLASTed against miRNAs of other species. (**A**) Number of conserved miRNAs in different species. (**B**) The statistics of occurrence numbers for conserved miRNA family members in different species.

To analyse the conservation of identified miRNAs, small RNA sequences were searched among vertebrate (51 species) using BLAST in miRBase v.21, four miRNAs (let-7, miR-97, miR-125 and miR-133) were conserved among more than 40 species, 48 miRNAs were conserved among more than 30 species, and 201 miRNAs were conserved among more than 20 species (Supplement Files: Table S4). MiRNA conservation among different species was analysed by aligning the sequences to known mammalian miRNAs, and the results indicated that the largest number of miRNAs were identified in *Homo sapiens*, with 1,515 miRNAs, followed by *Mus musculus* and *Bos taurus*, with 920 and 724 miRNAs, respectively, and 34 species were identified with more than 100 conserved miRNAs. The number of conserved miRNAs in different species is shown in Fig. 4A.

For all miRNA families, 772 miRNA families were identified in two libraries, and the number of miRNAs varied among different families (Fig. 4B), showing 6 families with over 100 members and 159 families with 10 to 100 members. The largest family was miR-2284 with 447 members, which was detected in 43 species, followed by let-7, miR-30, miR-143, miR-10 and miR-378; 152 families were the smallest, each containing only one member.

### miRNAs differential expression and putative target genes prediction

**Figure 5:**
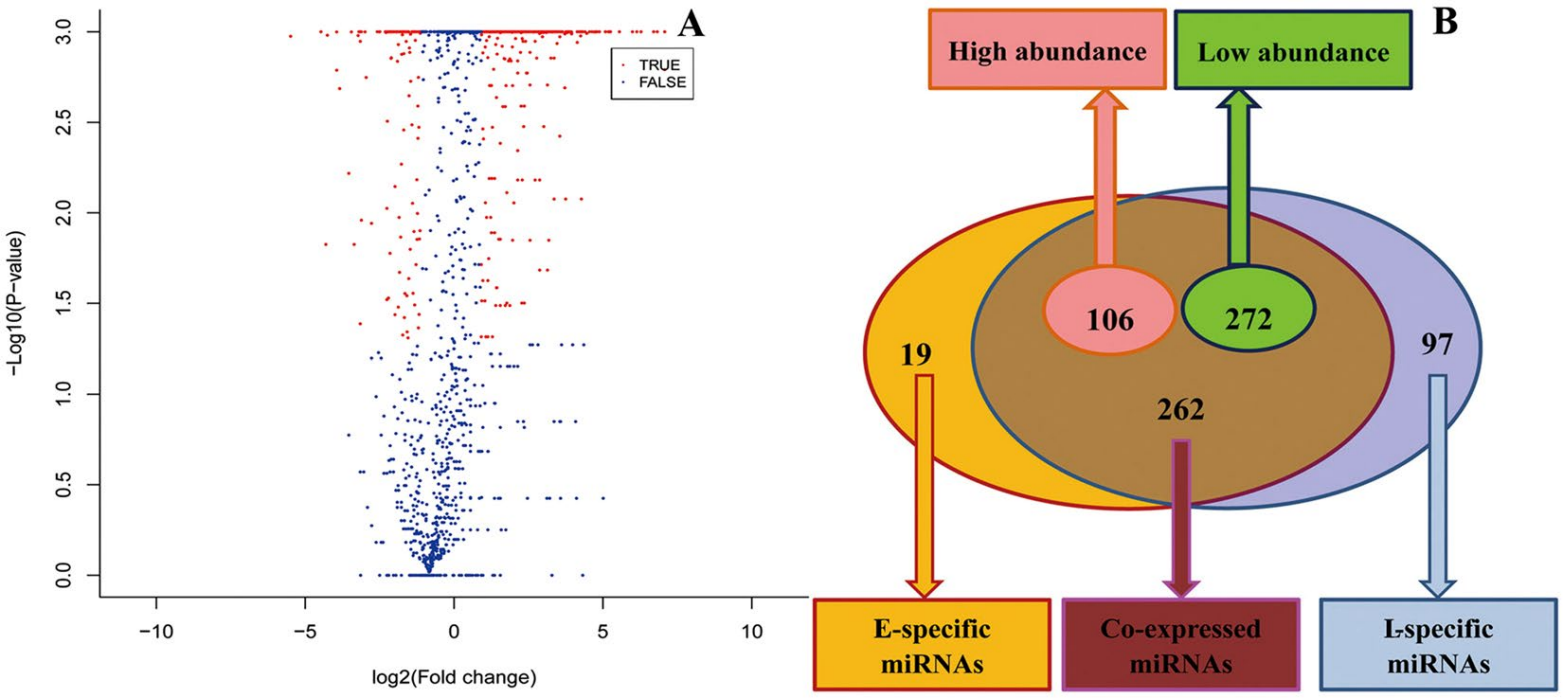
Differentially expressed miRNAs identified in early lactation (**E**) and late lactation (**L**) libraries. (**A**) The volcano diagram of differential expressed miRNAs in E and L libraries; blue dots represent miRNAs with no significant difference between two libraries, and red dots represent miRNAs with significant difference. (**B**) Venn diagram illustrating the distribution of 378 differential expressed miRNAs between E and L libraries, including 106 miRNAs with higher abundance and 272 miRNAs with lower abundance in E library compared to L library. The red circle shows the significant differentially expressed miRNAs in the E library, the blue circle shows the significant differentially expressed miRNAs in the L library, the cross section shows the co-expressed miRNAs, and the remaining molecules (yellow and blue) were specifically expressed miRNAs for the E and L libraries.

**Figure 6:**
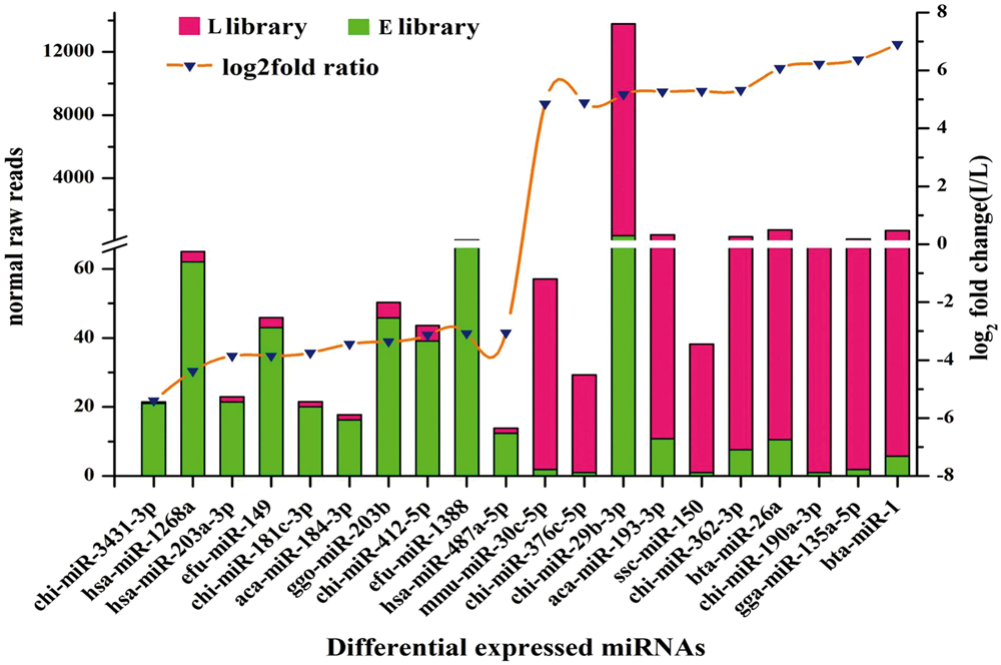
Reads and fold changes of the 20 most different miRNAs between early lactation (**E**) and late lactation (**L**) libraries. Green columns represent normal raw reads of each differentially expressed miRNA in the E library, red columns represent normal raw reads of each differentially expressed miRNA in the L library, the orange line represents the change trend of differentially expressed miRNAs between E and L libraries, and the blue triangles represent the value (log_2_^fold change (L/E)^) of each miRNA. The left Y-axis represents abundance (log_10_^(normal raw reads)^) of each miRNA in the E and L libraries. The right Y-axis represents the difference of relative expression abundance (log_2_^fold change (L/E)^) of each miRNA between the E and L libraries.

To further identify the characteristics of tissue-specific expression, all identified conserved miRNAs were analysed using Chi-squared and Fisher tests based on normalized high-throughput sequencing counts (Fig. 5 and Supplement Files: Table S5). The results showed the identification of 378 differentially expressed miRNAs with |log_2_(ratio)| ≥ 1 in early and late lactation, 315 differentially expressed miRNAs with *p* ≤ 0.01, 63 differentially expressed miRNAs with a *p* value from 0.05 to 0.01, and 262 miRNAs that were co-expressed. Compared to late lactation, 106 miRNAs had a higher abundance, and 19 miRNAs were specifically expressed in early lactation, while 272 had a higher abundance and 97 were specifically expressed in late lactation. The miRNA with biggest difference was bea-miR-1, with a 120.57-fold change, 6 and 463 raw reads in early and late lactation respectively, and four miRNAs (bta-miR-26a, chi-miR-362-3p, efu-miR-193-3p, and chi-miR-29b-3p) also showed higher differences (Fig. 6).

To obtain an overview of the functions of differentially expressed miRNAs, 287 miRNAs (log2ratio ≥ 1.5, *P* ≤ 0.05) were selected to predict their potential target genes using TargetScan and miRanda. In total, 32,458 target genes were obtained by TargetScan, 27,935 target genes were obtained by miRanda, and 10,452 non-redundant target genes were overlapped in two data sets (Supplement Files: Table S6). Among these differentially expressed miRNAs, chi-miR-455-3p has the most candidate target genes (1,900, accounting for 18.18% of 10,452 non-redundant target genes), each of 70 miRNAs has more than 1,000, 201 miRNAs had between 100 to 1,000 target genes, as each of 147 genes was targeted by more than 200 miRNAs, 430 genes were singly targeted by miRNAs between 100 to 200, and 2,109 genes were singly targeted by only one miRNA; the target gene with the largest number targeted by miRNAs was *CNTNAP2*, which was annotated with ATP binding and involved in fatty acid biosynthesis and the insulin signalling pathway.

### Go annotations and KEGG pathways analysis of candidate target genes

**Figure 7:**
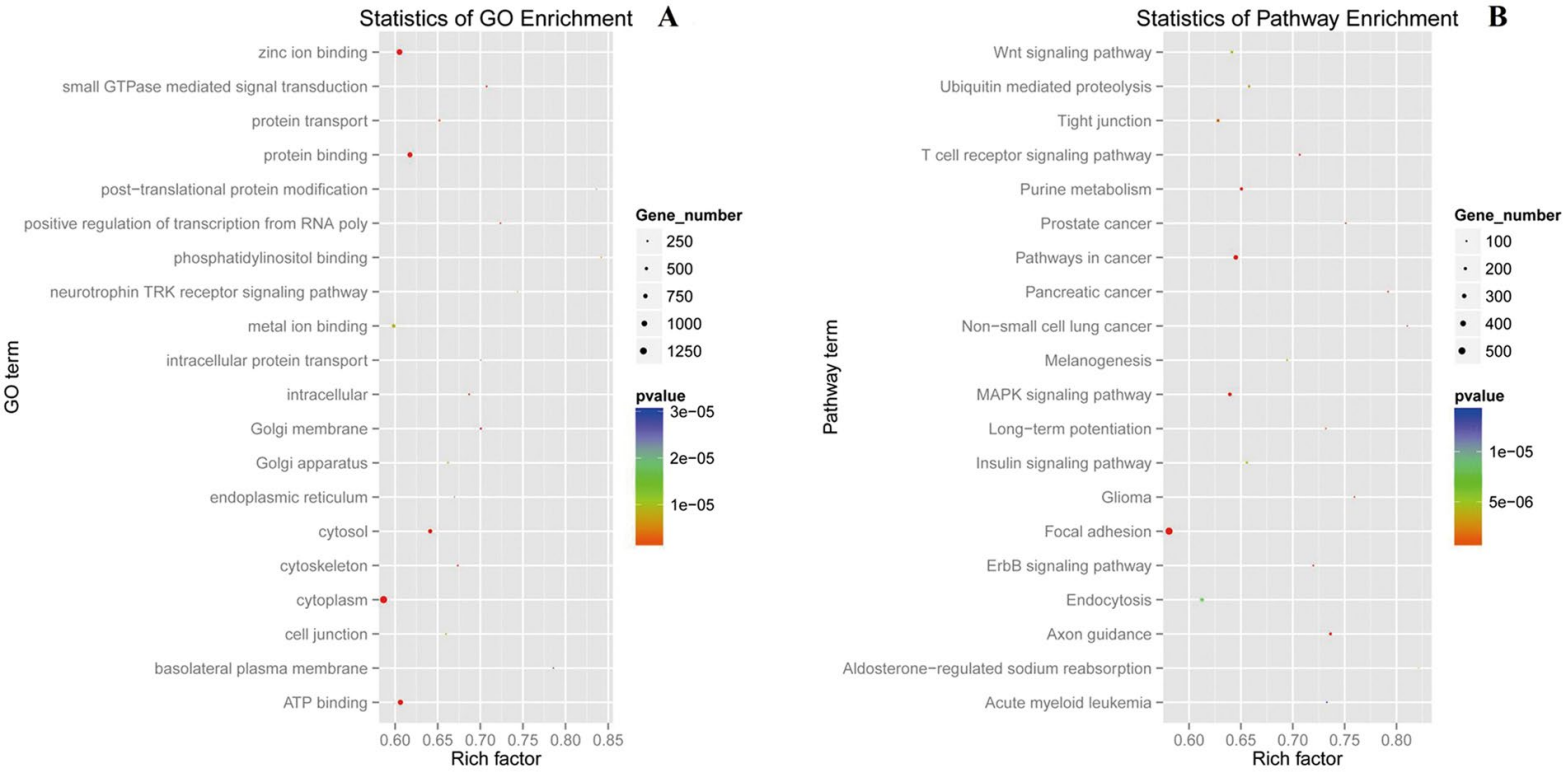
The statistics of GO and KEGG pathway enrichment. (**A**) the statistics of GO enrichment. (**B**) The statistics of KEGG pathway enrichment. The circlular dots represent the numbers of annotated genes for different GO (KEGG) terms. Different colours represent different levels of significance (*P*-value). The rich factor is a ratio of the gene numbers of each term to the numbers of all genes with terms.

To understand more about the roles of differentially expressed miRNAs between E and L libraries, 10,452 putative target genes targeted by 287 differentially expressed miRNAs were selected and submitted to Gene Ontology (GO) enrichment and Kyoto Encyclopaedia of Genes and Genomes (KEGG) analysis; GO annotation analysis showed that a total of 7,076 candidate target genes were annotated with 347 non-redundant terms, of which 3,324 candidate target genes were distributed into 181 Go terms based on biological process, 4,924 candidate target genes were distributed into 74 Go terms based on cellular component, and 92 Go terms for 4,452 candidate target genes were distributed into molecular component (Supplement Files: Table S6). Among all Go terms, the biggest is GO:0005634, annotated for 1,576 candidate target genes, and for all candidate target genes, the highest number was observed for tumour protein p63 (*TP63*), which was distributed into 2,340 Go terms; most of the genes were located in the nucleus and cytoplasm, related to binding activity, transport, development, signal transduction and other biological functions. The statistics of the enriched GO categories for the target genes of 287 differentially expressed miRNAs are shown in Fig. 7A and Supplement files: Table S7.

KEGG pathway analysis showed that 3,143 candidate target genes were grouped into 33 pathways, where *SKAP1* (src kinase-associated phosphoprotein 1) has the most pathways involved (15 pathways), followed by *IGF-1* (insulin-like growth factor I), *ITPR2* (inositol 1,4,5-trisphosphate receptor, type 2) and *MCPH1* (microcephaly 1), and the most enriched pathway was focal adhesion (Ko04510), with 501 (11.75%) annotated genes distributed into this group, followed by ECM-receptor interaction (Ko04512), cancer pathways (Ko05200), and the MAPK signalling pathway (Ko04010), with 370 (8.68%), 303 (7.11%) and 230 (5.39%) annotated genes, respectively. The statistics of the enriched pathways are shown in Fig. 7B and Supplement files: Table S8.

### miRNAs-Genes network construction

**Figure 8:**
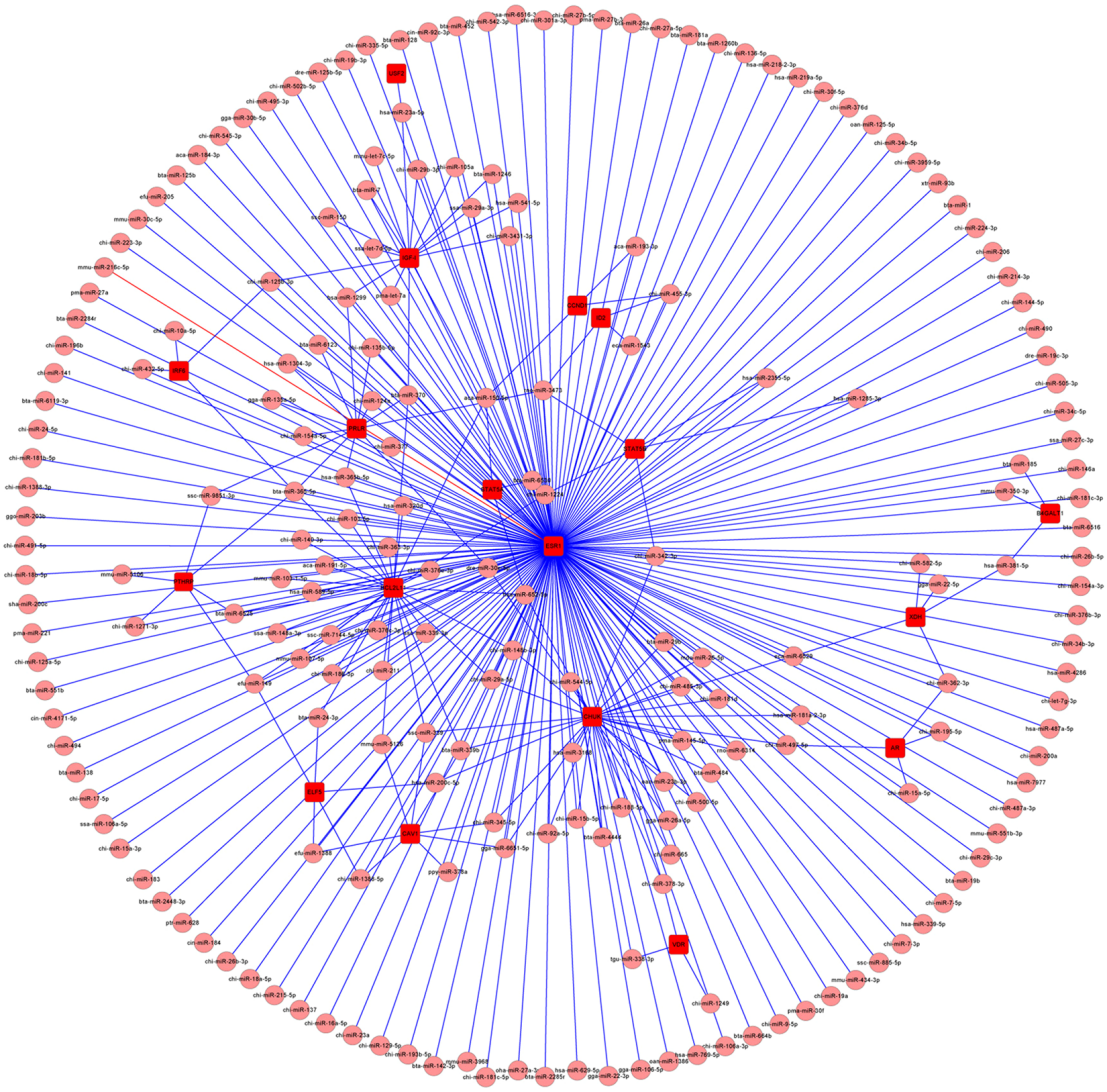
Predicted regulation relationships between different expressed miRNAs and target genes based on GO annotation. 214 differentially expressed miRNAs and 18 target genes annotated in mammary gland development/lactation were selected to construct the network, comprising 232 nodes (miRNA and gene) and 335 edges (regulatory relationship between miRNAs and target genes). The square frame with red colour presents target genes, the pink circle presents miRNAs, and the blue line presents a regulatory relationship between miRNAs and target genes.

**Figure 9:**
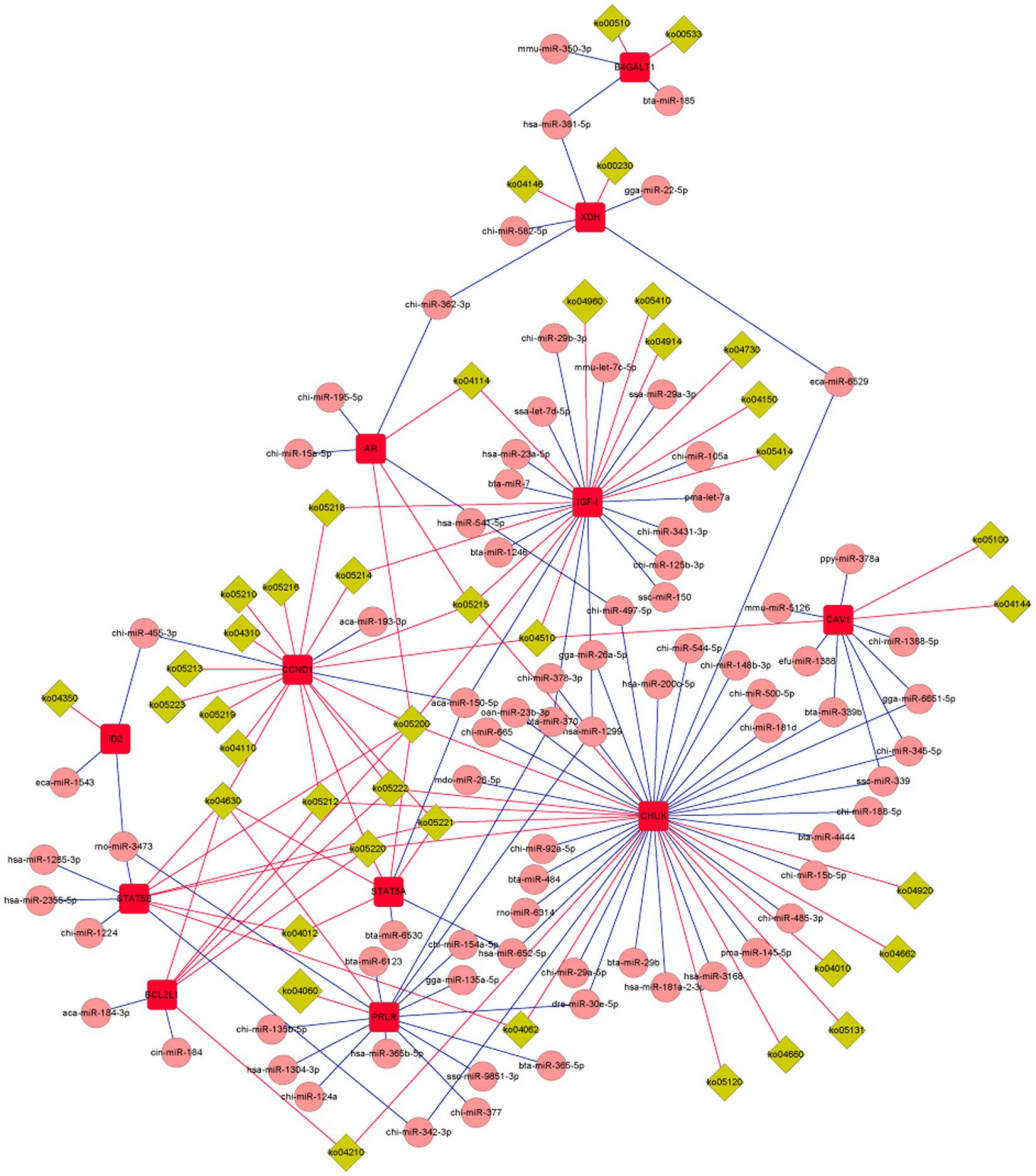
Predicted regulation relationships between differentially expressed miRNAs and target genes based on KEGG pathway. 79 differentially expressed miRNAs, 12 target genes annotated in mammary gland development/lactation and 41 KEGG pathways were selected to construct the network, comprising 132 nodes and 170 edges. The square frame with red colour presents target genes, the pink circle presents miRNAs, the green rhombus presents KEGG pathway, the blue line presents a regulatory relationship between miRNAs and target genes, and the red line presents the target genes involves in the KEGG pathway.

To identify the functional regulations from miRNAs to mRNAs, 18 predicted target genes, *STAT5B* (signal transducer and activator of transcription 5B), *STAT5A* (signal transducer and activator of transcription 5 A), *CHUK* (conserved helix-loop-helix ubiquitous kinase), *VDR* (vitamin D receptor), *CAV1* (caveolin 1), *PTHRP* (parathyroid hormone-like protein), *PRLR* (prolactin receptor), *ESR1* (oestrogen receptor 1), *USF2* (upstream transcription factor 2), *AR* (androgen receptor), *BCL2L11* (BCL2-like 11), *CCND1* (cyclin D1), *ELF5* (E74-like factor 5), *ID2* (inhibitor of DNA binding 2), *IRF6* (interferon regulatory factor 6), *XDH* (xanthine dehydrogenase), *IGF-I* and *B4GALT1* (UDP-Gal:betaGlcNAc beta 1,4- galactosyltransferase, polypeptide 1), directly annotated in mammary gland development and lactation, were selected to construct the networks. Based on their GO annotation and KEGG pathway analyses, a network with 232 nodes and 335 edges (Fig. 8) and a network with 132 nodes and 170 edges were constructed, respectively (Fig. 9); as shown in the network, multiple genes were simultaneously regulated by the same miRNA, and the core miRNAs and the centralized control gene function were identified, with 7 miRNAs (miR-150-5p, miR-365-5p, miR-339, miR-370, miR-625-5P, miR-149 and miR-3473) exhibiting important regulatory functions in these networks and more than four genes predicted as regulated by a single miRNA. miR-150-5p and miR-365-5p had five targeted genes involved in mammary gland development. Four genes, (*ESR1*, *CCND1*, *CHUK* and *IGF-1*) may be critical in mammary gland development: *ESR1* was regulated by most miRNAs, *CCND1* was involved in 17 pathways, *CHUK* and *IGF-1* were involved in 14 and 12 pathways, respectively, and three pathways (Ko05200: pathway in cancer, Ko05520: chronic myeloid leukaemia and Ko04630: Jak-STAT signalling pathway) may be valuable in mammary gland development; the numbers of genes involved in each pathway were seven, five and five, respectively.

## Discussion

MicroRNAs (miRNAs) represent a class of small RNA regulators encoded by a diverse range of eukaryotic organisms with important roles in many biological processes, including development, cell proliferation, differentiation and apoptosis, oncogenesis, and immune defence^19,30^. In recent years, high-throughput sequencing technology has shown special advantages and has been widely used in the identification and differential expression of miRNAs in various species^3–32^, enlarging the realm of miRNA research. The mammary gland, as a unique female organ that undergoes growth, development, degradation and other cyclical processes, plays a critical role in the synthesis and secretion of milk, neonatal nutrition acquisition and development needs, while the numbers, morphology and motility of mammary epithelial cells are different at different development stages, particularly for different lactation stages in the lactation circle, which involves the start of lactation, early lactation, peak lactation, late lactation and involution, and are accompanied by proliferation, differentiation and apoptosis of mammary epithelial cells; these steps in turn effect the synthesis, secretion, and yield of milk^1,3^. Although many studies have been implemented in mammary gland physiology via high-throughput sequencing technology^33–35^, the definite mechanism of miRNA, as a class of important regulators in mammary gland physiology, is remain unclear, particularly for different mammary gland interior environments, such as early and late lactation. In previous studies, two sRNA libraries in early and late lactation were constructed and sequenced, and the differentially expressed miRNAs between these two stages were revealed; the mechanism of miRNA regulation in mammary gland development was preliminarily discussed via target gene prediction, GO annotation and enrichment analysis, KEGG pathway analysis, and the construction of a regulatory network, which will provide a theoretical foundation for better understanding mammary gland physiology.

The sequencing data from two libraries revealed that 91.52% and 79.31% of the sequences were validated reads, and mRNAs only accounted for 0.45% and 0.62% (Table 1), suggesting a high sequencing quality in library construction and sequencing, and the overall distribution pattern of different length sequences was similar (Fig. 2). The small RNAs of 22 nt dominated, followed by 21 nt, 22 nt and 23 nt, consistent with previous reports on mammalian small RNAs distribution^36–38^, while a certain difference existed between the two libraries in the abundance of small RNAs lengths, implying the tissue-specific expression of small RNAs in early and late lactation. Considering the conservation of miRNAs among different species, we selected all mammalian miRNAs in miRBase v.21 as a background database, and to minimize the flash positive results, reads with more than three were used to identify the known and novel miRNAs, a total of 1,160 pre-miRNA and 1,442 unique miRNAs were identified in two libraries, and 758 miRNAs were co-expressed (Table 2 and Fig. 5). For miRNA families, each of 6 families (let-7, miR-2284, miR-30, miR-143, miR-10 and miR-378) had more than 100 members, and Alsaweed also reported the members of each family with higher expression abundance^39^. In addition, the same miRNA family member in two libraries showed some difference in expression abundance; these results implied that these family members performed an important regulatory role in mammary gland development. To further certify the classification of identified miRNAs, six types of classification methods were based on the alignments of sequences with miRNAs, pre-miRNAs and genomes; 397 unique miRNAs were named as known miRNAs (Group 1a) in *Capra hircus*, 1,045 unique miRNAs and 912 pre-miRNAs were identified as conserved miRNAs among mammals, and 45 sequences with free energy ranging from −141.8 kcal/mol to −17.7 kcal/mol, and hairpin structures precursors were named as novel miRNA candidates (Table 2 and Table S3).

The typical characteristic of miRNAs is the tissue- and spatiotemporal-specific expression patterns. To determine the relationship and difference between the two libraries, expression analysis was performed to identify the conserved and differentially expressed miRNAs. We observed that *Homo sapiens* has the largest number of conserved miRNAs, followed by *Mus musculus* and *Bos taurus*; 201 miRNAs were conserved among more than 20 species (Fig. 4A and Supplement Files: Table S4), 106 miRNAs had a higher abundance, 272 had a lower abundance, and 19 were specifically expressed in early lactation compared to late lactation. Twenty miRNAs showing the most significant difference were all middle or highly expressed (Figs 5 and 6 and Table S5). High-abundance expressed miRNAs identified in the present study, including miR-375, miR-10, miR-26, miR-29, and miR-126, were also identified in other mammalian mammary gland tissues, even in the milk and body fluids^40–42^. We concluded that these miRNAs might play important regulatory roles in different physiological development stages of dairy goat mammary glands.

miRNAs perform critical regulatory roles by targeting different mRNAs; for elucidating the biological functions of miRNAs to obtain their presumptive target genes, two crucial methods (TargetScan and MiRanda) were employed to predict their putative target genes in our study, and we observed that the same gene was targeted by different miRNAs and that a single miRNA exerted regulatory effects on a number of target genes. For example, 1,900 target gene candidates were predicted for miR-455-3p, miR-339-5p has 1,975 putative target genes, *TG* (thyroglobulin) was targeted by 122 miRNAs, and *FOXP2* (forkhead box P2) was targeted by 106 miRNAs. These results indicated that miRNAs performed complex functions by regulating different genes in mammary gland development, and this was also clear by GO annotation and KEGG pathway analyses. This finding is also indicative of the limitation of the current methods, and further experiments and analyses are needed to confirm accurate relationships and functions of these miRNAs and their target genes.

GO annotation and KEGG pathway analyses are critical for better understanding miRNA functions and have been viewed as promising methods for uncovering the miRNA gene regulatory network. In the present study, 7,076 putative target genes of 287 differential expressed miRNAs were annotated in GO consortium, 3,324 genes were annotated in biological processes, 4,924 were in cellular component, and 4,453 were in molecular function (Supplement Files: Tables S6 and S7). For molecular functions, the binding terms and related binding terms comprised most of the targeted genes, consistent with a regulatory role for these miRNAs in the transcription and translation processes^43^. In addition, KEGG analysis showed signal miRNAs involved in different pathways and many miRNAs involved in the same pathway, indicating the functional complexity of miRNA (Supplement Files: Table S8).

## Materials and Methods

### Ethics approval and informed consent

All animal experiments were approved by the Institutional Animal Care and Use Ethics Committee of Shandong Agricultural University and performed in accordance with the “Guidelines for Experimental Animals” of the Ministry of Science and Technology (Beijing, China), and all efforts were made to minimize suffering.

### Animal sample preparation and total RNA extraction

The Laoshan dairy goat is one of four Chinese dairy goat breeds, with a long history of cultivation, genetic stability and other excellent germplasm characteristics. In the present study, five Laoshan dairy goats (four-year-old, third lactation period) from Laoshan dairy goat primary farm in Qingdao, Shandong Province, China, were used. These animals were healthy without disease and reared in pens in the same feeding and management conditions.

The mammary gland tissues from the same side at different development stages of early lactation (10^th^ day after parturition) and late lactation (210^th^ day after parturition) were collected by surgery (general anaesthesia by intramuscular injection of xylazine hydrochloride) immediately after milking and frozen immediately in liquid nitrogen for further use. Total RNA was extracted from the above ten mammary gland tissues using TRIzol reagent (Invitrogen, Carlsbad, USA) according to the manufacturer’s instructions, and the quantity and integrity were analysed and assessed using an Agilent 2100 Bioanalyser (Agilent Technologies, USA).

### Small RNA library construction and sequencing

RNA samples from five mammary gland tissues at the same stage were equivalently mixed and homogenized for each small RNA library construction, named the E library (early lactation) and L library (late lactation), respectively. The experimental procedures were strictly performed according to the recommended protocols (Illumina Inc., USA) with TruSeq^TM^ Small RNA Sample Prep Kits (Illumina, San Diego, USA). The general processes are as follows: 10 μg of total RNA was used, and 18–30 nt fragments were isolated and purified using 15% denaturing polyacrylamide gel electrophoresis (PAGE). Subsequently, RNA 3′ and 5′ adapters were ligated to the RNA pool using T4 RNA ligase 2 and subjected to reverse transcription polymerase chain reaction (RT-PCR) to obtain the single-strand cDNA for further PCR amplification. Finally, the amplification products of 140~160 bp were purified, validated and sequenced.

### Sequencing data processing

Sequencing data was processed using ACGT101-miR v4.2 data analysis soft. Briefly, the analysis was conducted using the following protocol: after being extracted from the image data, the raw reads were assessed using Illumina FastQC to obtain the Q30 high-quality data. Further, a series of digital filters were employed to remove the adapter dimers, junk and low complexity sequences, the remaining sequences (also named as clean reads) were aligned to mRNA, Rfam and Repbase database to further discard mRNA, other ncRNAs (rRNA, tRNA, snRNA, snoRNA), and repeat sequences, the un-mappable sequences were used for miRNAs identification, and according to their alignment on miRBase (release v.21) and genome (*Capra hircus* v1.01), these reads were classified into six groups: Reads mapped to miRNAs/pre-miRNAs of *Capra hircus* in miRbase and the pre-miRNAs further mapped to the genome & EST were classified as Group 1a; Reads mapped to mammalian (except for *Capra hircus*) miRNAs/pre-miRNAs in miRbase and the pre-miRNAs further mapped to the genome & EST were classified as Group 1b; Reads mapped to mammalian miRNAs/pre-miRNAs in miRbase, the mapped pre-miRNAs, which were not further mapped to the genome, and the reads (and of course the miRNAs of the pre-miRNAs) mapped to the genome and the extended genome sequences from the genome loci that may form hairpins were classified as Group 2a; Reads mapped to mammalian miRNAs/pre-miRNAs in miRbase, the mapped pre-miRNAs, which were not further mapped to the genome, and the reads (and of course the miRNAs of the pre-miRNAs) mapped to the genome and the extended genome sequences from the genome loci that may not form hairpins were classified as Group 2b; Reads mapped to mammalian miRNAs/pre-miRNAs in miRbase, the mapped pre-miRNAs not further mapped to the genome, and the reads not mapped to the genome but mapped to the miRNAs (matures) were classified as Group 3. Reads that were not mapped to mammalian pre-miRNAs in the miRBase but were mapped to the genome and EST of *Capra hircus* and the extended genome sequences that may form hairpins were named Group 4.

### Identification of known and novel miRNAs

Unique sequences with lengths of 18–26 nt were subsequently mapped to the precursor sequences of mammalian miRNAs in the miRBase by BLAST searching to identify known miRNAs and novel 3p- and 5p- derived miRNAs. According to their alignments with miRNAs, pre-miRNAs and genomes, the reads were classified into six categories (please refer to Sequencing data processing in Materials and Methods). Group 1a included known miRNAs of *Capra hircus*, Group 1b, Group 2 and Group 3 included conserved miRNAs among *Capra hircus* and other mammals, and Group 4 included novel miRNAs. The hairpin RNA structures containing sequences were predicted from the flanking 80-nt sequences using RNAfold software (http://rna.tbi.univie.ac.at/cgi-bin/RNAfold.cgi). The criteria for secondary structure prediction were: (1) number of nucleotides in one bulge of the stem ≤ 12; (2) number of base pairs in the stem region of the predicted hairpin ≥ 16; (3) cut-off free energy (kCal/mol) ≤ −15; (4) length of hairpin (up and down stem + terminal loop) ≥ 50; (5) length of hairpin loop ≤ 20; (6) number of nucleotides in one bulge in the mature region ≤ 4; (7) number of biased errors in one bulge in the mature region ≤ 2; (8) number of biased bulges in the mature region ≤ 2; (9) number of errors in the mature region ≤ 4; (10) number of base pairs in the mature region of the predicted hairpin ≥ 12; and (11) percentage of mature regions in the stem ≥ 80.

### miRNAs abundance and differential expression

miRNA expression was first normalized to obtain the expression of transcripts per million (TPM) for better visualization and comparability, and the fold-change and *P*-value were calculated from the normalized expression using the following formulas:

Normalized expression = (Actual miRNA sequencing reads count/Total clean reads count) × 1,000,000. Fold change = Log2 (L /E), *P*-value:

$${\rm{P}}({\rm{x}}|{\rm{y}})=(\frac{{N}_{2}}{{N}_{1}})\frac{(x+y)}{x!y!{(1+\frac{{N}_{2}}{{N}_{1}})}^{(x+y+1)}}\begin{array}{c}C(y\le {y}_{{\rm{\min }}}|x)=\sum _{y=0}^{y\le {y}_{{\rm{\min }}}}p(y|x)\\ D(y\ge {y}_{{\rm{\max }}}|x)=\sum _{y\ge {y}_{{\rm{\max }}}}^{\infty }p(y|x)\end{array}$$

The expression levels were compared between the E library and L library; if the normalized expression value of a given miRNA is zero, then the expression value was modified to 0.01. If the normalized expression of a given miRNA was less than 1 in both libraries, then it was removed in future differential expression analyses. miRNAs with more than 10 reads, a *p* value below 0.05 and showing a fold change (log2) greater than 1.5 were considered differentially expressed. Finally, the significance of each library was tested with Fisher’s exact test and chi-square test.

### Target genes prediction, GO enrichment and KEGG pathway analysis

TargetScan and MiRanda online software were used to predict the potential target genes of differentially expressed miRNAs, the data predicted by both algorithms were combined, and the intersection elements were accepted as candidate target genes.

InterProScan (http://www.geneontology.org/GO.annotation.interproscan.shtml)^44^ and Blast2go (http://www.blast2go.com/b2ghome)^45^ were used to execute GO annotation and enrichment analysis from three ontologies: molecular function, cellular component and biological process. The GO terms were significantly enriched in the predicted target gene candidates of the miRN compared with the reference gene background and the genes corresponding to certain biological functions. The GO terms with corrected *P*-values ≤ 0.5 are defined as significantly enriched in the target gene candidates.

Cytoscape software V3.2.1 (http://www.cytoscape.org/)^46^ and the ClueGO plug-in (http://apps.cytoscape.org/apps/cluego) were used to decipher the KEGG (Kyoto Encyclopaedia of Genes and Genomes, http://www.genome.jp/kegg/)^47^ pathway and understand their biological functions. The genes with *P* ≤ 0.5 were considered significantly enriched in target gene candidates.

### Regulation network construction

To elucidate the regulation relationships from miRNAs to mRNAs, a subset of miRNAs and target genes was selected to construct their networks and visualized using Cytoscape v2.8 software-based GO annotation and KEGG pathway analyses, respectively.

### qRT-PCR validation of know and novel miRNAs

Quantitative real-time PCR was performed using the Mx3000p^TM^ SYBR^®^ Green real-time quantitative PCR Analyser (Stratagene, USA), Mx3000/Mx Pro-software was used to construct a melting curve, standard curves with 5-fold dilutions were performed for each assay, and the PCR efficiency calculations were based on the slopes of the standard curves. All reactions were performed in triplicate, and each sample was replicated three times. The primers were designed according to the instruction (Mir-X™ miRNA qRT-PCR SYBR^®^ Kit, Clontech), and appropriate modifications were made in the 5′ terminus; all primers of miRNAs are listed in the Table S1 in Supplement files. Each miRNA level was expressed as 2^−ΔΔCT^ mean ± SE (standard error), one-way ANOVA was used to examine the significance of differential expression level in each mature/novel miRNA between early and late lactation, and the difference was considered significant when *P* ≤ 0.05. The housekeeping gene *U6* was used as an endogenous control for the normalisation analyses.

### Data availability statement

All data generated or analysed in the present study are included in this published article (and its Supplementary Information files).

## Electronic supplementary material

Supplementary info file

Table S1

Table S2

Table S3

Table S4

Table S5

Table S6

Table S7

Table S8
