# Supplementary material for: Screening of miRNA profiles and construction of regulation networks in early and late lactation of dairy goat mammary glands
Source: Sci Rep. 2017 Sep 20;7:11933. doi: 10.1038/s41598-017-12297-4 (PMC5607250; doi:10.1038/s41598-017-12297-4)
Supplement: Supplementary file 1 — Supplementary info file [file 41598_2017_12297_MOESM1_ESM.pdf]

Screening of miRNA profiles and constructing of regulation network in early and late lactation of dairy goat mammary gland

Zhibin Ji, Zhaohua Liu, Tianle Chao, Lei Hou, Rui Fan, Rongyan He, Guizhi Wang*, Jianmin Wang*

Shandong Provincial Key Laboratory of Animal Biotechnology and Disease Control and Prevention, College of Animal Science and Veterinary Medicine, Shandong Agricultural University, Taian 271018, Shandong Province, P.R. China

[*] **Correspondence author:**

Jianmin Wang

E-mail: wangjm@sdau.edu.cn

Tel: +86-538-8241448

Fax: +86-538-8241419

Guizhi Wang

E-mail: wangjm@sdau.edu.cn

Tel: +86-538-8241448

Fax: +86-538-8241419

Supplementary Information

Table S1: The related information of miRNAs validated by qRT-PCR.

Table S2: All conserved and known miRNAs identified in two libraries.

Table S3: All novel miRNAs identified in two libraries.

Table S4: Conservation of the identified miRNAs among different species.

Table S5: 378 differentially expressed miRNAs between early and late lactation libraries.

Table S6: Target genes and GO annotation of 287 differentially expressed miRNA.

Table S7: The statistic of the enriched GO categories for 7,076 candidate target genes.

Table S8: The statistic of the enriched KEGG pathway for 3,143 candidate target genes.
