# Supplementary material for: Screening of miRNA profiles and construction of regulation networks in early and late lactation of dairy goat mammary glands
Source: Sci Rep. 2017 Sep 20;7:11933. doi: 10.1038/s41598-017-12297-4 (PMC5607250; doi:10.1038/s41598-017-12297-4)
Supplement: Supplementary file 2 — Table S1 [file 41598_2017_12297_MOESM2_ESM.doc]

Table S1: The related information of miRNAs validated by quantitative real-time polymerase chain reaction.

| miRNA name | Sequence in miRBase  (5’→3’) | Primer sequence  (5’→3’) | Length (nt) | GC% | Tm |
| --- | --- | --- | --- | --- | --- |
| chi-miR-29b-3p | TAGCACCATTTGAAATCAGT | CGTAGCACCATTTGAAATCAGTG | 23 | 43.5 | 61.8 |
| chi-miR-23a | ATCACATTGCCAGGGATTTCC | ATCACATTGCCAGGGATTTC | 20 | 45.0 | 59.8 |
| chi-miR-451-5p | AAACCGTTACCATTACTGA | GGCAAACCGTTACCATTACTGA | 22 | 45.5 | 61.1 |
| chi-miR-26b-5p | TTCAAGTAATTCAGGATAGGTT | CGCTTCAAGTAATTCAGGATAGG | 23 | 43.5 | 59.3 |
| chi-miR-100-5p | AACCCGTAGATCCGAACTTGT | AACCCGTAGATCCGAACTTGT | 21 | 47.6 | 59.9 |
| chi-miR-99b-5p | CACCCGTAGAACCGACCTTGCG | ATACCCGTAGAACCGACCTTGCG | 23 | 56.5 | 67.3 |
| chi-miR-10a-5p | TACCCTGTAGATCCGAATTTGT | GTACCCTGTAGATCCGAATTTG | 22 | 45.5 | 57.6 |
| Novel-PC-3p-4922 |  | ATATCGGAACCTGCGGATAC | 20 | 50.0 | 58.9 |
| Novel-PC-3p-13184 |  | ATAGGAACATGGACTCTGGGC | 21 | 52.4 | 60.3 |
| Novel-PC-5p-3196 |  | AAGCTGGAGACTACTGAAGTGAC | 23 | 47.8 | 56.9 |
